# Knowledge and attitudes of medical and pharmacy university students regarding monkeypox: a multicenter, cross-sectional study in Vietnam

**DOI:** 10.1186/s12909-024-05805-4

**Published:** 2024-07-29

**Authors:** Dung Anh Doan, Thuy Thi Xuan Nguyen, Giang Ba Le, Trinh Lan Phuong, Phuong Lan Nguyen, Dai Xuan Dinh

**Affiliations:** 1 Faculty of Pharmacy, Phenikaa University, Hanoi, Vietnam; 2 Department of Pharmacy, Da Nang University of Medical Technology and Pharmacy, Da Nang, Vietnam; 3 Faculty of Public Health, Pham Ngoc Thach University of Medicine, Ho Chi Minh, Vietnam; 4 Drug Administration of Vietnam, Ministry of Health, Hanoi, Vietnam; 5 Faculty of Pharmaceutical Management and Economics, Hanoi University of Pharmacy, Hanoi, Vietnam

**Keywords:** Attitude, Knowledge, Mpox, Monkeypox, Medical student, Pharmacy student, University student, Vietnam

## Abstract

**Background:**

In May 2022, monkeypox (mpox) suddenly reappeared and rapidly spread worldwide. This global outbreak was a public health emergency of international concern. This study investigated Vietnamese medical and pharmacy university students’ knowledge and attitudes towards mpox.

**Methods:**

This online cross-section survey was administered among students in four universities using a convenience sampling method. A semi-structured questionnaire was shared among students via a Google Forms link (quick-response code) in university amphitheaters at recess. Frequency (percentage) and mean (standard division) were used for descriptive statistics. Univariate and multivariate linear regression analyses were conducted to identify factors associated with students’ mpox knowledge and attitudes. A *p*-value < 0.001 was considered statistical significance.

**Results:**

A total of 1,848 students participated in this study (including 1,030 medical students and 818 pharmacy students). Their common sources for seeking mpox/health information included the Internet (89.7%) and mass media (64.2%). Students’ average knowledge and attitude scores were 11.542 ± 3.910 (range: 0–23) and 30.301 ± 3.738 (range: 9–45), respectively. The average knowledge score of pharmacy students (12.139 ± 3.545) was significantly higher than that of medical students (11.067 ± 4.118) (*p* < 0.001). The average attitude score of the former (30.454 ± 3.787) was comparatively higher than that of the latter (30.181 ± 3.696), but this difference was insignificant (*p* = 0.111). Factors significantly associated with students’ mpox knowledge and/or attitudes were their sex, age, year of study, residence, major, grade point average, type of university, seeking mpox information in the past, and using reliable sources to seek health information (*p* < 0.001). A positive correlation existed between students’ knowledge and attitude scores (*p* < 0.001).

**Conclusions:**

Students’ knowledge about mpox was relatively poor, while their attitudes were comparatively positive. Universities and relevant authorities should focus on the above factors and use multiple platforms and channels of communication to guarantee that trustworthy information about mpox can reach as many students as possible.

## Background

Monkeypox (mpox) is a viral disease caused by the mpox virus – a species of the genus Orthopoxvirus. Physical contact with infected people/animals or contaminated materials is the main way of mpox transmission [1]. In May 2022, an outbreak of mpox suddenly reappeared and rapidly spread worldwide, especially in Europe and the Americas. Most new cases were from countries without previously documented mpox transmission [2]. In July 2022, the World Health Organization (WHO) declared that this global outbreak of mpox was a public health emergency of international concern [1]. As of 22 August 2023, there were 89,529 mpox confirmed cases and 156 deaths globally. About 114 countries reported cases of mpox. Six countries with the highest numbers of mpox cases included the United States (30,534), Brazil (10,967), Spain (7,565), France (4,150), Colombia (4,090), and Mexico (4,050). Most cases were men (96.3%), men aged 18–44 years old (79.3%), and men who have sex with men (82.8%). Sexual encounters were the most common form of transmission (82.5%). From May to August 2023, the Western Pacific Region, the Americas, and Africa were the most affected regions. On average, there have been 150 observed cases per week worldwide. Up to August 2023, the number of mpox cases in Vietnam was negligible (two cases in October 2022 and one in July 2023) [2].

In May 2023, the WHO announced that this mpox outbreak did not constitute a public health emergency of international concern anymore. However, mpox has still been a topic of interest. Instead of using emergency measures, there has been a need to have long-term strategies to manage and control the public health risks posed by mpox [3]. As per The International Health Regulations Emergency Committee, several current matters of concern included the low level of mpox knowledge and the shortage of capacities in diagnosis, medicines, and vaccines in many countries, especially in low-income countries. Albeit a decline in mpox cases globally, this virus has continued to be transmitted to specific communities. Hence, countries should maintain their surveillance, control measures, preparedness, and response capacities, thereby preventing future outbreaks [3]. Having community engagement in the battle against mpox outbreaks is of paramount importance.

Healthcare university students are young, energetic, and enthusiastic. With the ability to quickly grasp information and news from education, social networks, and media, university students can play an essential role in educating the general population and contributing to combat epidemics and pandemics. However, only several studies have been conducted to measure healthcare students’ knowledge and attitudes towards mpox worldwide [4–7]. High proportions of students lacking necessary knowledge and having negative attitudes towards mpox were reported. There is a paucity of information about the knowledge and attitudes of healthcare university students in Vietnam. This study was conducted to investigate medical and pharmacy university students’ knowledge and attitudes towards mpox in Vietnam in 2023.

## Methods

### Study design and setting

This was a multicenter cross-sectional quantitative study. University students were approached from May to June 2023. Vietnam can be divided into three regions: northern, central, and southern. Two universities in the northern part, one in the central part, and one in the southern part were purposively selected for this study. Data were collected from four universities where the researchers worked, including the Hanoi University of Pharmacy, the Phenikaa University, the Da Nang University of Medical Technology and Pharmacy, and the Pham Ngoc Thach University of Medicine.

### Participants and sample size

The subjects were Vietnamese undergraduate students pursuing a Bachelor of Medicine or a Bachelor of Pharmacy. Inclusion criteria included those studying in the four above universities, willing to participate in this research, and able to read and understand the Vietnamese language. The Raosoft Sample Size Calculator was used to compute the study sample size. The minimum sample size was 665 students (with a confidence level: 99%, a margin of error: 5%, and a response distribution: 50%). Participants were recruited using a convenience sampling method. The research team endeavoured to approach as many students as possible to increase the reliability, generalization, and extrapolation of results.

### Study procedure

A semi-structured questionnaire was designed via Google Forms. The first page included a short introduction about the study aims, researchers’ declaration of anonymity and confidentiality, and students’ confirmation of voluntary participation. A Google Form link with the questionnaire was transformed into a quick-response (QR) code. The research team showed this code in university amphitheaters at recess and invited students to answer the questionnaire. The participants could access the questionnaire by scanning the QR code with their smartphones or other electronic gadgets. The questionnaire (the Google Form link) was also distributed via social networks (Facebook groups of the four universities above) to increase the sample size.

### Variables and measurement

#### Independent variables

The personal information of the participants was collected, including sex (male/female), year of birth, region (northern/central/southern), area (urban/rural), marital status (married/unmarried), type of university (public/private), year of study (first/second/third/fourth/fifth/sixth), major (medical/pharmacy), grade point average (GPA) for the last semester, monthly allowance and income (million Vietnam dongs), seeking mpox information in the past (yes/no), and sources of information about mpox and other health information.

#### Dependent variables

Two primary outcomes were students’ knowledge and attitudes towards mpox. The research team developed a questionnaire through a literature review [2, 4–31]. The knowledge part (Table 1) consisted of 23 items about mpox (including its epidemiology, modes of transmission, symptoms, treatment, prevention, and vaccination). Three answering options were True, False, and Do not know. The attitude part (Table 2) included nine items using a 5-point Likert scale (answering options: Totally disagree, Disagree, Neutral/Normal, Agree, and Totally agree). Students were asked about their feelings or thoughts about mpox, their willingness to accept isolation, and beliefs in self-protection and the ability to control and conquer the pandemic if outbreaks occur.

**Table 1:** The mpox knowledge of medical and pharmacy university students in Vietnam (*N* = 1,848 students)

**Knowledge items**	**Average score (Standard deviation)**		
	**Medical student**	**Pharmacy student**	**Total**
1. Mpox is a disease infection caused by a newly discovered virus in 2022 (False)	0.381 (0.486)	0.385 (0.487)	0.383 (0.486)
2. Mpox is prevalent in Western and Central Africa (True)	0.761 (0.427)	0.746 (0.436)	0.754 (0.431)
3. There were hundreds of mpox confirmed cases in Vietnam in 2022 (False)	0.430 (0.495)	0.515 (0.500)	0.468 (0.499)
4. The mpox virus circulates only among primates, such as monkeys and humans (False)	0.270 (0.444)	0.331 (0.471)	0.297 (0.457)
5. Most of the mpox confirmed cases are men (True)	0.298 (0.458)	0.259 (0.438)	0.281 (0.450)
6. The mortality rate of mpox is about 50% (False)	0.336 (0.473)	0.454 (0.498)	0.388 (0.487)
7. Mpox could be transmitted through blood (No evidence = False)	0.176 (0.381)	0.145 (0.353)	0.162 (0.369)
8. The virus can spread during pregnancy (from the mother to the fetus) (True)	0.574 (0.495)	0.659 (0.474)	0.611 (0.488)
9. Mpox can be transmitted by close contact with lesions, bodily fluids, respiratory droplets, and materials contaminated with the virus (True)	0.852 (0.355)	0.889 (0.315)	0.869 (0.338)
10. The virus can survive for several days on contaminated surfaces (True)	0.641 (0.480)	0.605 (0.489)	0.625 (0.484)
11. The typical incubation period of the mpox virus is about 5—21 days (True)	0.676 (0.468)	0.707 (0.456)	0.689 (0.463)
12. The mpox virus causes milder illness in children than adults (False)	0.506 (0.500)	0.581 (0.494)	0.539 (0.499)
13. Mpox and smallpox do not have similar signs and symptoms (False)	0.418 (0.494)	0.445 (0.497)	0.430 (0.495)
14. Swollen lymph nodes (lymphadenopathy) are one clinical sign or symptom that could be used to differentiate mpox and smallpox cases (True)	0.585 (0.493)	0.674 (0.469)	0.624 (0.484)
15. The common symptoms of mpox include fever, headache, rash, and swollen lymph nodes (True)	0.825 (0.380)	0.862 (0.345)	0.841 (0.365)
16. Diarrhea, convulsions, and loss of smell are the common symptoms of mpox (False)	0.229 (0.420)	0.323 (0.468)	0.271 (0.444)
17. Using hand sanitizers, gloves, and masks plays a role in reducing the risk of catching mpox (True)	0.857 (0.350)	0.901 (0.299)	0.877 (0.329)
18. There is a vaccine against mpox (True)	0.328 (0.470)	0.377 (0.485)	0.350 (0.477)
19. There is a treatment approved specifically for mpox virus infections (False)	0.233 (0.423)	0.293 (0.456)	0.260 (0.439)
20. One management option for symptomatic mpox patients is to use paracetamol (True)	0.534 (0.499)	0.538 (0.499)	0.536 (0.499)
21. Antibiotics can be used to treat mpox (False)	0.309 (0.462)	0.422 (0.494)	0.359 (0.480)
22. People who received chickenpox vaccine are immunized against mpox (False)	0.476 (0.500)	0.572 (0.495)	0.518 (0.500)
23. A smallpox vaccine can be used for mpox (True)	0.372 (0.484)	0.458 (0.499)	0.410 (0.492)
**Average knowledge score**	11.067 (4.118)	12.139 (3.545)	11.542 (3.910)
Good (≥ 50% of the maximum score)	542 (52.6)	505 (61.7)	1,047 (56.7)
Poor (< 50% of the maximum score)	488 (47.4)	313 (38.3)	801 (43.3)

**Table 2:** The mpox attitudes of medical and pharmacy university students in Vietnam (*N* = 1,848 students)

**Attitude items**	**Average score (Standard deviation)**		
	**Medical student**	**Pharmacy student**	**Total**
1. I am very interested and want to learn more about mpox (Positive)	3.712 (1.183)	3.781 (0.985)	3.742 (1.100)
2. I am scared and worried about mpox (Negative)	2.618 (1.041)	2.534 (1.033)	2.581 (1.038)
3. If infected with mpox, I will be devastated (Negative)	2.825 (1.052)	2.760 (1.062)	2.797 (1.056)
4. I have negative feelings towards mpox that it might become a worldwide pandemic like COVID-19 (Negative)	2.982 (1.003)	2.950 (1.022)	2.968 (1.011)
5. If outbreaks occur, Vietnam can control and win the battle against mpox (Positive)	3.525 (0.952)	3.661 (0.868)	3.585 (0.918)
6. The global population can control mpox worldwide (Positive)	3.386 (0.930)	3.441 (0.884)	3.411 (0.910)
7. I can protect myself against mpox if there is an outbreak (Positive)	3.558 (0.904)	3.562 (0.859)	3.560 (0.884)
8. When an outbreak occurs, even if I have another disease, I will not go to the hospital for treatment because there is a risk of catching mpox there (Negative)	3.577 (1.114)	3.650 (1.024)	3.609 (1.075)
9. If infected with mpox, I will accept isolation to avoid spreading mpox to others (Positive)	3.997 (1.027)	4.112 (1.014)	4.048 (1.022)
**Average attitude score**	30.181 (3.696)	30.454 (3.787)	30.301 (3.738)
Positive (≥ 50% of the maximum score)	908 (88.2)	713 (87.2)	1,621 (87.7)
Negative (< 50% of the maximum score)	122 (11.8)	105 (12.8)	227 (12.3)

Three senior lecturers reviewed questions to assess the face validity of the questionnaire. After that, a pilot study with the participation of 40 students was conducted to check the clarity of each question and assess its reliability. The good level of internal consistency of this questionnaire (for the knowledge and attitude parts) was demonstrated through Cronbach’s alpha (0.91 and 0.89), split-half reliability (0.960 and 0.872), and Omega Total (0.93 and 0.93), respectively. To assess the test–retest reliability, the above students answered the questionnaire one more time (two weeks after the first survey). Their average knowledge and attitude scores slightly increased, but the differences were insignificant (knowledge score: 9.800 ± 4.286 and 10.300 ± 3.764, *p* = 0.581; attitude score: 31.250 ± 5.457 and 31.600 ± 4.727, *p* = 0.760). Intraclass correlation coefficients (ICC) higher than 0.75 (knowledge part: ICC = 0.934 (95%CI: 0.901–0.960), attitude part: ICC = 0.887 (95%CI: 0.829–0.932)) also proved the good test–retest reliability of this questionnaire at two different times of measurement.

### Data analysis

R software (version 4.3.1) was employed to analyze data. Numbers with percentages and means ± standard deviations (SDs) were used for descriptive statistics. To avoid missing values, in Google Forms, the required toggle buttons were enabled to make all questions mandatory questions. Unless all questions were answered, the participants could submit their responses. Therefore, there were no missing values in the data.

The knowledge and attitude scores of each student were computed. For each knowledge item, one score was for a correct answer, while 0 was for a “Do not know” or incorrect answer. The score for each attitude item ranged from one (strongly negative) to five (strongly positive). Students’ possible knowledge and attitude scores were from 0–23 and 9–45, respectively. Higher scores indicated better knowledge and more positive attitudes towards mpox. In this study, a 50% cut-off point was used to categorize students’ knowledge and attitude scores, similar to many previous studies [32–35]. The Wilcoxon rank-sum test was used to compare the knowledge and attitude scores between the medical and pharmacy student groups since the data were not normally distributed. For numeric variables, data distribution was assessed via histograms and the Shapiro–Wilk test. Factors associated with students’ knowledge and attitudes towards mpox were determined through univariate and multivariate linear regression models. The Bayesian Model Averaging method was employed to select dependent variables in the multivariate models to minimize the complexity of models and avoid overfitting and multicollinearity. A *p*-value < 0.001 was considered statistical significance.

## Results

### General characteristics of participants

**Fig. 1:**
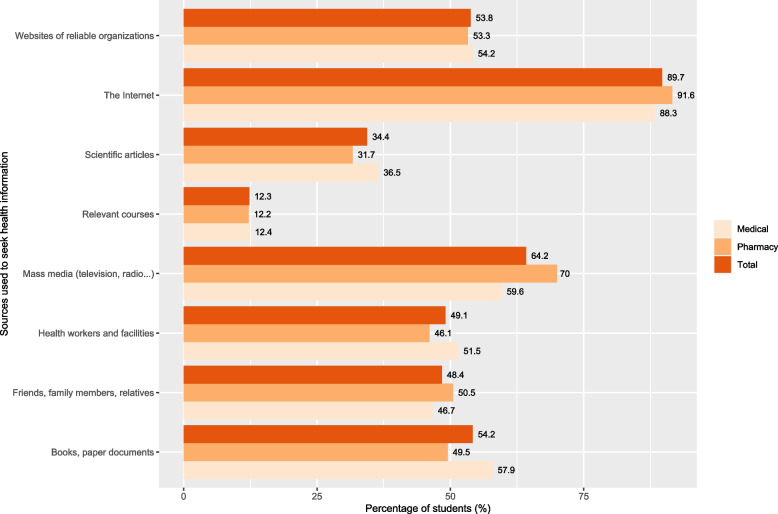
Common sources that Vietnamese medical and pharmacy university students used to seek mpox and health information

A total of 1,877 students answered the first question of the questionnaire (Do you agree to take part in this survey?). Twenty-nine students selected the “No” answer. About 98.5% (1,848 students) concurred to participate in this research. The number of medical and pharmacy university students was 1,030 and 818, respectively. Nearly two-thirds of participants were female (62.9%). Their average age was 21.264 ± 1.659 years old. Only 2.1% got married. More than 70% of them were studying in public universities. More than half came from northern Vietnam (55.1%) and urban areas (56.8%). Most students (89.7%) had a monthly allowance and income of less than six million Vietnam dongs (252.89US$). Approximately 45.2% of students previously sought information about mpox (Table 3). Common sources that students used to seek mpox and health information included the Internet (89.7%), mass media (64.2%), and books/paper documents (54.2%). The proportions of students reading scientific articles and participating in relevant courses were low (34.4% and 12.3%, respectively) (Fig. 1).

**Table 3:** The characteristics of the study sample (*N* = 1,848 students)

**Characteristics**		**Number (%)**		
		**Medical student**	**Pharmacy student**	**Total**
Sex	Male	465 (45.1)	221 (27.0)	686 (37.1)
	Female	565 (54.9)	597 (73.0)	1,162 (62.9)
Age (years old)	19	246 (23.9)	145 (17.7)	391 (21.2)
	20	145 (14.1)	111 (13.6)	256 (13.9)
	21	200 (19.4)	204 (24.9)	404 (21.9)
	22	75 (7.3)	159 (19.4)	234 (12.7)
	23	258 (25.0)	174 (21.3)	432 (23.4)
	24 or higher	106 (10.3)	25 (3.1)	131 (7.1)
Marital status	Married	24 (2.3)	14 (1.7)	38 (2.1)
	Unmarried	1,006 (97.7)	804 (98.3)	1,810 (97.9)
Region (Residence)	Northern	251 (24.4)	768 (93.9)	1,019 (55.1)
	Central	248 (24.1)	33 (4.0)	281 (15.2)
	Southern	531 (51.6)	17 (2.1)	548 (29.7)
Area	Urban	616 (59.8)	433 (52.9)	1,049 (56.8)
	Rural	414 (40.2)	385 (47.1)	799 (43.2)
Type of university	Public	729 (70.8)	576 (70.4)	1,305 (70.6)
	Private	301 (29.2)	242 (29.6)	543 (29.4)
Year of study	First	307 (29.8)	151 (18.5)	458 (24.8)
	Second	120 (11.7)	104 (12.7)	224 (12.1)
	Third	219 (21.3)	219 (26.8)	438 (23.7)
	Fourth	42 (4.1)	161 (19.7)	203 (11.0)
	Fifth and sixth	342 (33.2)	183 (22.4)	525 (28.4)
Monthly allowance and income (million Vietnam dongs) Exchange rate: 1 million Vietnam dongs = 42.15US$	< 3	530 (51.5)	452 (55.3)	982 (53.1)
	3 to < 6	376 (36.5)	300 (36.7)	676 (36.6)
	6 to < 9	82 (8.0)	38 (4.6)	120 (6.5)
	9 or higher	42 (4.1)	28 (3.4)	70 (3.8)
Grade point average (GPA) for the last semester (scale: 0–4)	2.50 or lower	257 (25.0)	158 (19.3)	415 (22.5)
	2.51 to 3.00	396 (38.4)	379 (46.3)	775 (41.9)
	3.01 to 3.50	302 (29.3)	212 (25.9)	514 (27.8)
	3.51 to 4.00	75 (7.3)	69 (8.4)	144 (7.8)
Seeking mpox information in the past	Yes	438 (42.5)	397 (48.5)	835 (45.2)
	No	592 (57.5)	421 (51.5)	1,013 (54.8)

### Vietnamese medical and pharmacy university students’ knowledge and attitudes towards mpox

**Fig. 2:**
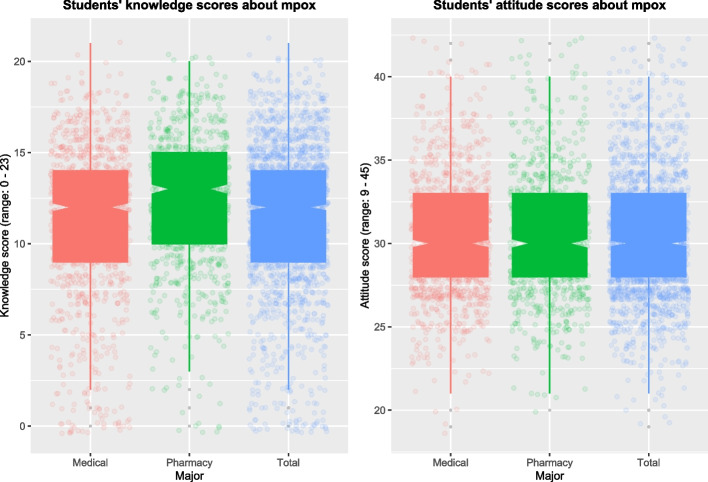
Distribution of the knowledge and attitude scores of Vietnamese medical and pharmacy university students

In general, Vietnamese university students had an acceptable level of knowledge about the prevalence, main modes of transmission, incubation period, symptoms, and prevention. However, only 16.2% of students understood that the mpox virus was yet to know to be able to be transmitted through blood. About a quarter (28.1%) knew that most mpox confirmed cases were men. Although most students (84.1%) knew fever, rash, and swollen lymph nodes were the common mpox symptoms, only a few knew diarrhea, convulsions, and loss of smell were not (27.1%). Regarding mpox vaccination, about a third were aware that there was at least one vaccine against mpox and antibiotics could not be used to treat this virus. The average knowledge score of all 1,848 students was 11.542 ± 3.910. The average knowledge score of pharmacy students (12.139 ± 3.545) was significantly higher than that of medical students (11.067 ± 4.118) (*p* < 0.001). The proportion of students who had good knowledge of mpox was 56.7% (Table 1, Fig. 2).

Regarding students’ attitudes towards mpox, many students felt scared and worried about mpox (883 students, 47.7%). About 38.5% (712 students) would be devastated if catching this virus. Nearly a third (32.6%) thought mpox could become a worldwide pandemic like the Coronavirus disease 2019 (COVID-19). In case of mpox outbreaks, even if catching another disease, about 16.4% of students would not go to the hospital for treatment due to the risk of catching mpox there. However, many believed that Vietnam could win the battle against mpox (58.3%) and that the global population could control mpox worldwide (49.1%). Approximately 80.7% (1,492 students) accepted isolation to avoid spreading mpox to other people if infected with this virus. Nearly two-thirds (65.4%) wanted to learn more about mpox. In general, the mpox attitudes of students were relatively positive. The average attitude score of all students was 30.301 ± 3.738. The average score of medical students (30.181 ± 3.696) was comparatively lower than that of pharmacy students (30.454 ± 3.787), but this difference was insignificant (*p* = 0.111) (Table 2, Fig. 2).

### Factors associated with the mpox knowledge and attitudes of Vietnamese medical and pharmacy university students

There were positive correlations between students’ knowledge scores and their age (*p* < 0.001), attitudes and age (*p* < 0.001), and knowledge and attitude scores (*p* < 0.001). Medical students' knowledge and attitude scores were significantly lower than those of pharmacy students (*p* < 0.001). Students from the south had lower knowledge scores and higher attitude scores than those from other region groups (*p* < 0.001). Year of study (*p* < 0.001), GPA (*p* < 0.001), seeking mpox information in the past (*p* < 0.001), and using reliable sources to seek health information (such as scientific articles and websites of WHO/MOH) were also significantly associated with students’ knowledge and attitudes towards mpox (*p* < 0.001). In addition, in comparison with males, female students had higher knowledge scores (*p* < 0.001). Other factors associated with students’ positive attitudes towards mpox included studying in a private university (*p* < 0.001) and receiving health information from health workers and facilities (*p* < 0.001) (Table 4).

**Table 4:** Factors associated with the mpox knowledge and attitudes of healthcare university students in Vietnam (*N* = 1,848 students)

**Independent variables**		**Knowledge score**				**Attitude score**			
		**Univariate**		**Multivariate**		**Univariate**		**Multivariate**	
		**coef**	***p*-value**	**a.coef**	***p*-value**	**coef**	***p*-value**	**a.coef**	***p*-value**
**Sex** (ref: Female)	Male	-0.894	**< 0.001**	-0.480	0.006	0.443	0.014		
**Age (years old)** (continuous variable)		0.182	**< 0.001**			0.220	**< 0.001**		
**Marital status** (ref: Married)	Unmarried	-0.871	0.174			-1.707	0.005		
**Region (Residence)** (ref: Central)	Northern	0.037	0.886	0.824	**< 0.001**	0.982	**< 0.001**		
	Southern	-1.291	**< 0.001**			0.990	**< 0.001**	1.297	**< 0.001**
**Area** (ref: Rural)	Urban	-0.100	0.587			0.147	0.401		
**Type of university** (ref: Private)	Public	-0.005	0.980			-1.146	**< 0.001**	-1.617	**< 0.001**
**Major** (ref: Medical)	Pharmacy	1.072	**< 0.001**			0.273	0.119	0.703	**< 0.001**
**Year of study** (ref: Fifth and sixth)	First	-1.175	**< 0.001**	-0.687	**< 0.001**	-1.322	**< 0.001**	-0.735	**< 0.001**
	Second	-0.463	0.134			-1.038	**< 0.001**		
	Third	0.574	0.022	0.629	0.002	-0.801	**< 0.001**		
	Fourth	-0.204	0.523			-0.729	0.017		
**Grade point average (GPA) for the last semester** (scale: 0–4) (ref: 2.50 or lower)	2.51 – 3.00	1.833	**< 0.001**	1.464	**< 0.001**	0.787	**< 0.001**		
	3.01 – 3.50	2.849	**< 0.001**	2.382	**< 0.001**	0.991	**< 0.001**		
	3.51 – 4.00	3.584	**< 0.001**	3.023	**< 0.001**	1.226	**< 0.001**		
**Monthly allowance and income** (ref: 3 to < 6) (million Vietnam dongs)^a^	< 3	-0.369	0.059			-0.460	0.013		
	6 to < 9	0.135	0.727			0.992	0.007		
	9 or higher	0.816	0.096			0.406	0.385		
**Seeking mpox information in the past** (ref: No)	Yes	2.147	**< 0.001**	1.792	**< 0.001**	0.857	**< 0.001**	0.492	0.004
**Sources used to seek mpox and health information**									
**Books, paper documents** (ref: No)	Yes	0.172	0.347			0.354	0.043		
**The Internet** (ref: No)	Yes	0.234	0.434			-0.409	0.153		
**Mass media** (ref: No)	Yes	0.280	0.140			0.309	0.088		
**Friends, family members, relatives** (ref: No)	Yes	-0.109	0.550			-0.198	0.255		
**Health workers and facilities** (ref: No)	Yes	0.218	0.231			0.774	**< 0.001**		
**Websites of reliable organizations** (ref: No)	Yes	0.890	**< 0.001**			1.298	**< 0.001**	0.731	**< 0.001**
**Scientific articles** (ref: No)	Yes	0.907	**< 0.001**	0.724	**< 0.001**	1.232	**< 0.001**	0.830	**< 0.001**
**Relevant courses** (ref: No)	Yes	0.473	0.087			0.001	0.996	-0.884	0.001
**Knowledge score**						0.164	**< 0.001**	0.128	**< 0.001**

Adjusted R-squared for the multivariate linear regression models: 0.1751 (Knowledge) and 0.1025 (Attitude)

*Coef* coefficient, *a.coef* adjusted coefficient, *ref* reference

^a^ Exchange rate: 1 million Vietnam dongs = 42.15US$

## Discussion

This cross-sectional study was conducted to investigate the knowledge and attitudes towards mpox among medical and pharmacy university students in Vietnam. The results showed that the Internet and mass media were students' most common sources for seeking mpox and health information. Students’ knowledge about mpox was relatively poor, while their attitudes were comparatively positive. Many students lacked knowledge about the epidemiology, mortality rate, ways of transmission, vaccination, and mpox treatment. Many students were worried about mpox and would be devastated if coming down with this disease. The main factors significantly associated with both students’ knowledge and attitudes towards mpox included their age, GPA, year of study, major, residence, seeking mpox information in the past, and using reliable sources to seek health information (such as scientific articles and websites of WHO/MOH). In addition, male students had lower mpox knowledge scores than females. The attitudes towards mpox among students from a private university and those receiving health information from health workers and facilities were more positive than other groups. There is a need to enhance Vietnamese medical and pharmacy university students’ knowledge about mpox if mpox outbreaks occur in the future.

Vietnamese university students’ knowledge about mpox was relatively poor. About 43.3% of students had knowledge scores lower than 11.5 (50% of the maximum score), far lower than the result of a study in Saudi Arabia (72%) [6]. A possible reason is that data were collected in the middle of 2023, while the latter was from May to July 2022. In the United Arab Emirates (UAE), 22.8%, 57.3%, and 19.9% of university students had good, moderate, and poor mpox knowledge, respectively (Bloom’s cut-off point). In Vietnam, these percentages were 1.2%, 33.0%, and 65.8%, respectively. A rationale behind the high level of knowledge about mpox among students in UAE was that a high proportion of UAE students received information about human mpox during education [4]. In Vietnam, information about mpox was mainly disseminated via social networks and media for a relatively short time. Besides university students, previous studies revealed that low and moderate levels of knowledge about mpox were also witnessed among Chinese men who have sex with men [16], the Lebanese population [24], clinicians and the general public in the United States [18, 23], medical students and clinical doctors from 17 Arab countries [21], and healthcare workers in Italy, Algeria, and China [22, 30, 36]. In case of mpox outbreaks in the future, the government and authorities need to have practical solutions and campaigns to enhance the mpox knowledge for not only university students but also other population groups.

Negative attitudes towards mpox among healthcare personnel and the public were a matter of concern in many countries [18, 24, 31, 37]. In Pakistan, 48.6% of the general population thought that mpox was a fatal disease. 38.3% agreed that this virus could cause a pandemic like COVID-19 [14]. In China, 62.7% of people were concerned about mpox outbreaks [10]. 56.7% of medical workers were apprehensive about this virus [13]. In 11 Arabic countries, nearly two-thirds of healthcare workers and medical students were worried about the risk of catching mpox and the increasing number of mpox cases that could lead to a national lockdown [26]. The apprehension about the capacity that mpox can become a new pandemic was also reported in a study with the participation of 11,919 medical students in 27 countries [5]. In this study, besides several negative feelings and perturbation, a high proportion of Vietnamese university students believed that the global population could win the battle against mpox and would accept isolation if mpox outbreaks occurred, similar to the results of studies in Saudi Arabia [7] and Bangladesh [15]. More importantly, a majority of university students said that they wanted to learn more about mpox, demonstrating their inquisitive spirit and studiousness (Vietnam: 65.4%, Saudi Arabia: 71.0% [6], 27 countries across three continents: 91.8% [5]). This can facilitate the development of educational interventions and campaigns involving mpox in the future.

In Saudi Arabia, higher knowledge scores were found among medical students with higher age, year of study, and GPA [6]. In UAE, older age, females, being infected with chickenpox, and receiving mpox information during education were the main factors associated with students’ mpox knowledge [4]. In Vietnam, besides sex, age, year of study, and GPA, other factors significantly associated with students’ knowledge and/or attitudes towards mpox included major, residence, type of university, seeking mpox information in the past, and information sources. Using reliable sources to seek mpox and health information (such as scientific articles and websites of WHO/MOH) could help students avoid misinformation, disinformation, and fake news during the era of cutting-edge technology and the Internet boom [38]. In addition, students with higher age, year of study, and GPA might have more experience in identifying trustworthy sources of information. Therefore, they could have better knowledge and attitude scores. By way of illustration, in this study, in comparison with younger students (first, second, and third years of study), the percentages of older students (fourth year of study or higher) using credible information sources were higher (for example, websites of reliable organizations such as WHO/MOH: 48.9% and 61.3%, scientific articles: 30.4% and 40.5%, respectively).

Regarding sources of information used to seek mpox and health information, the findings of a cross-sectional study with the participation of medical students from 27 countries showed that nearly three-quarters utilized social media to have information about mpox (73.7%), followed by scientific websites (50.6%) [5]. Medical students and clinical doctors from 17 Arab countries used social media (89.9%) and the Internet (82.7%) [21]. In UAE, university students received information about mpox via social media (64.5%) and television (27.4%) [4]. In Saudi Arabia, social networks and mass media were also the common sources of mpox information among medical students (Twitter: 62.1%, Snapchat: 48.1%, and television: 30.8%) [6, 7]. The Internet and social media were the two common information sources used by healthcare personnel and the general population to seek information about mpox and health [13, 19, 24, 26]. Meanwhile, more credible sources constituted a considerably smaller percentage of users (for example, research articles: 14.7% in Saudi Arabia and 16.9% in Arabic countries) [6, 26]. Raising awareness and educating the public to prevent mpox transmission and infection are crucial in controlling this disease. University students (the young generation) are more familiar with technology and the Internet. They can make a contribution to disseminating accurate health information and news to their friends, families, and other people. To prepare for the battle of future mpox outbreaks, universities, health organizations, and relevant authorities should use multiple platforms and channels of communication to guarantee that trustworthy information about mpox can reach as many people as possible, including healthcare students.

Regarding strengths, this is a multicenter study with a large sample of students. Using a cut-off of 0.001 for the *p*-value can contribute to ensuring the reproducibility of findings. Furthermore, the Bayesian Model Averaging method was employed to select independent variables in the multivariate linear regression models to reduce models’ complexity and prevent multicollinearity and overfitting. However, this study has several limitations. First, the causal relationships between students’ knowledge/attitudes towards mpox and independent variables could not be established because this was only a cross-sectional study. Second, we used a self-reported questionnaire to collect data, which can be subject to recall and information bias. Instead of having a probabilistic sample, we used convenience and snowballing sampling methods to recruit students, which can lower the generalization of the whole population. Finally, using electronic platforms to collect data could exclude students who did not use the Internet.

## Conclusions

Students’ knowledge about mpox was relatively poor, while their attitudes were comparatively positive. Factors significantly associated with students’ knowledge and/or attitudes towards mpox included their sex, age, year of study, average mark (GPA), major, residence, type of university, seeking mpox information in the past, and using reliable sources to seek mpox and health information.

## Data Availability

The datasets used and/or analyzed during the current study are available from the corresponding author upon reasonable request.

## Abbreviations

95%CI: 95% Confidence interval
GPA: Grade Point Average
ICC: Intraclass Correlation Coefficients
MOH: Ministry of Health
Mpox: Monkeypox
SD: Standard deviation
UAE: United Arab Emirates
WHO: World Health Organization
